# Ray Wu, fifth business or father of DNA sequencing?

**DOI:** 10.1007/s13238-016-0271-8

**Published:** 2016-06-15

**Authors:** Yu Xue, Yongbo Wang, Hui Shen

**Affiliations:** Department of Bioinformatics & Systems Biology, College of Life Science and Technology, Huazhong University of Science and Technology, Wuhan, 430074 China; Center for Epigenetics, Van Andel Research Institute, Grand Rapids, MI 49503 USA

**Figure 1:**
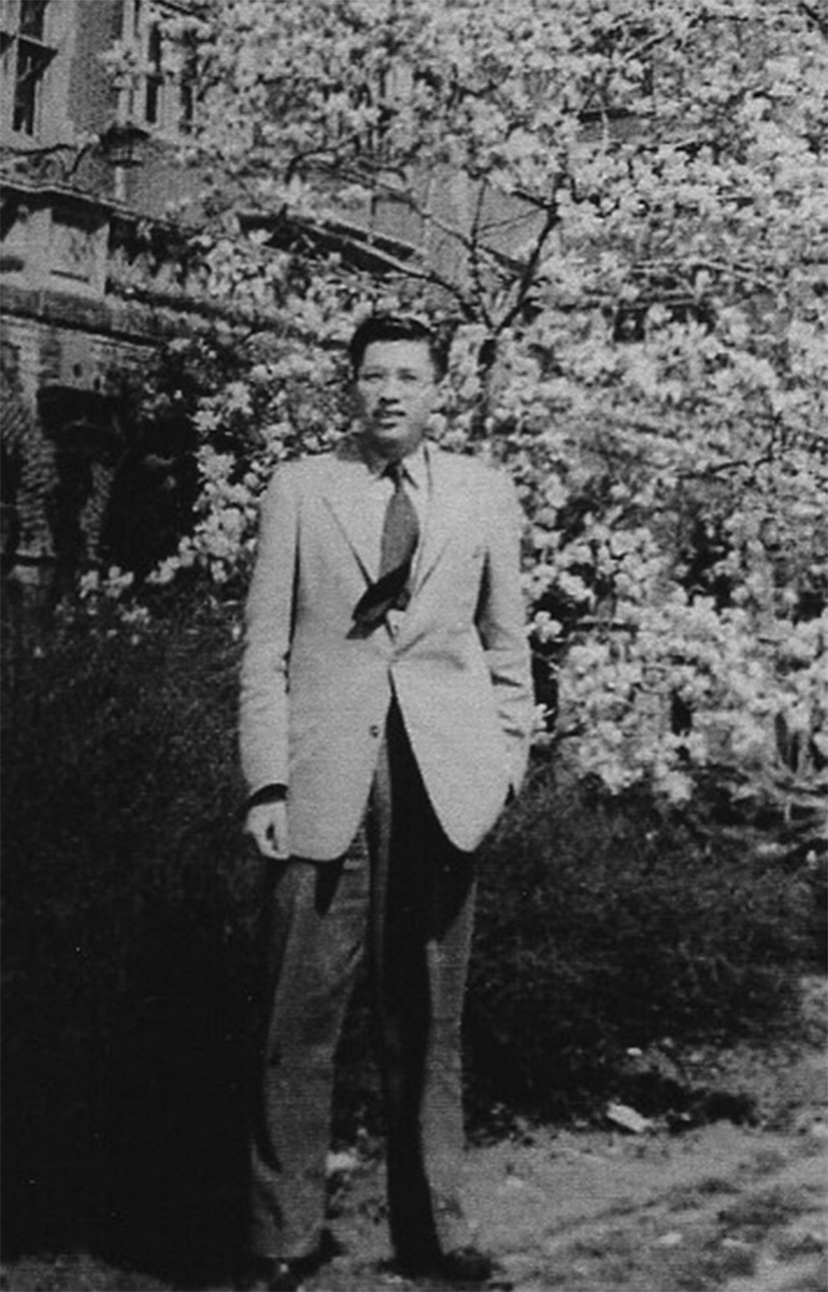
Dr. Ray Wu in the University of Pennsylvania in 1954

The first author, Yu Xue, first read about Dr. Wu in the ScienceNet blog written in 2008 by Prof. Yi Rao, an eminent Chinese neurobiologist, who has made a profound and lasting influence on the new generation of Chinese scientists, including us. Later, this essay was included in one of his published books, and we carefully read it again during the Spring Festival of 2015. One sentence was quite confusing to us, “The primer-extension approach developed by Ray Wu (Fig. 1) in 1971, is a key step of DNA sequencing, and deserves a Nobel Prize. At first we had to laugh at this comment and concluded that Prof. Rao must have been drunk or something when writing this, because all textbooks that we had been aware of stated Sanger sequencing as the first and most important methodology. On top of everything the Nobel Prize in Chemistry was awarded to Dr. Frederick Sanger and Dr. Walter Gilbert “for their contributions concerning the determination of base sequences in nucleic acids” in 1980, and we strongly doubted whether it would make sense to raise such seemingly meaningless sentiment. Also, in the traditional Chinese culture, understanding the true meaning of someone’s words can be quite tricky. For example, for grant application, a reviewer’s comment stating “this project MAY be funded” should be always interpreted as “this project can certainly NOT be funded”. Thus, we concluded that Prof. Rao was being sensationalist in his blog.

Generally, three major contributions of Dr. Wu have been widely recognized. The first and most well-known is the organization of the CUSBEA (China-United States Biochemistry Examination and Application, from 1981 to 1989) program. With this support, over four hundred excellent Chinese students got opportunities to pursue postgraduate studies in USA, achieved academic successes and have now largely become elite scientists in various fields (Gu, 2009). Second, Dr. Wu is deemed as “one of the founding fathers of plant genetic engineering” (Jiang, 2009), a field which looks wonderful but actually does not mean much to the public, since not many biologists in China work on plants. Third, one of Dr. Wu’s graduate students, Dr. Jack Szostak, won the Nobel Prize for Physiology or Medicine in 2009 for the discovery of telomeres (Szostak, 2009).

Let us start with the first aforementioned contribution, i.e., CUSBEA. Actually, we didn’t experience the CUSBEA, because when it was closed Yu Xue was only nine years old. And at his time of graduation from University of Science and Technology of China (USTC), students preferred to apply for scholarships of foreign universities to fund their training abroad. Nowadays, the scientific communication between China and other countries has become much more frequent, and people can go to study abroad either at their own expense, or supported by the government. So CUSBEA is a special occurrence in a unique historical period, and the new generation in current days never has the opportunity to experience it. So our conclusion is that recalling CUSBEA is just the “Recall past bitterness and appreciate sweetness at hand more” of the middle age generation, and youngers simply cannot relate. In addition, the participation for organizing CUSBEA belongs in the category of scientific and social service, and has nothing to do with Dr. Wu’s scientific contributions *per se*.

Second, the fact that one of Dr. Wu’s former students was awarded the Nobel Prize, was merely evidence that Dr. Wu was good at teaching. However, an intelligent student can be successful elsewhere, since there are a lot of great laboratories in the world. Thus, the student’s prize cannot firmly prove anything of Dr. Wu’s scientific achievements. Finally, Dr. Wu was usually recognized as “one of the founding fathers of plant genetic engineering” (Jiang, 2009), or a much smaller title “founding father of ABRC (Agricultural Biotechnology Research Center)”, an institute in Taipei (Yang and Lan, 2009). Since we are not experts in plants, we have no particular feelings towards it. And isn’t genetic engineering techniques usually first studied in animals, and then brought into plants? That makes it look like a less important contribution to us. In addition, Dr. Wu did not obtain any big awards, and was never elected to the National Academy of Science (NAS) in USA. So can we conclude that Dr. Wu contributed nil or almost nil for the science?

Yu Xue needed to teach a Bioinformatics course to undergraduate students, and found that his previous materials were quite dated and decided to update the course with more cutting-edge content. During his research he came across a book entitled of “Next-Generation DNA Sequencing Informatics” (Brown, 2013). This book had been translated into Chinese by Dr. Jun Yu et al. at the Beijing Institute of Genomics (BIG) as a textbook for introducing computational processes in analyzing the NGS data. In the first paragraph of Chapter 1, he found the statement “An interesting approximation of the Sanger method was published in 1971 by Ray Wu of Cornell University (Wu and Taylor, 1971)” which reminded him of the comments made by Prof. Yi Rao. Even budding yeasts and liver cancer cells in his lab knew that Dr. Sanger published the first paper introducing an efficient method for determining DNA sequences in 1975 (Sanger and Coulson, 1975), and two years later Dr. Gilbert developed a chemical procedure for DNA sequencing (Maxam and Gilbert, 1977). Then in 1977 Dr. Sanger greatly improved his strategy and developed the “Sanger Sequencing” approach, which earned him a Nobel Prize (Sanger et al., 1977). No other textbooks that he had read mentioned the contributions by Dr. Wu. So perhaps that Dr. Brown has made a mistake in his book?

So he decided to find the truth by doing a little bit research on PubMed. The literature showed several things. First, Dr. Wu was the first who attempted to develop the DNA sequencing approach, since his first paper on it was published in 1968 (Wu and Kaiser, 1968). Second, from 1968 to 1972, Dr. Wu’s group at least published 9 papers in formal journals for DNA sequencing (Donelson and Wu, 1972a, b; Padmanabhan and Wu, 1972a, b; Padmanabhan et al., 1972; Wu, 1970, 1972; Wu and Kaiser, 1968; Wu and Taylor, 1971). Third, Dr. Wu continued to publish three papers in 1973 and seven papers in 1974. So before the Sanger’s first paper on sequencing (Sanger and Coulson, 1975), Dr. Wu’s group at least published up to 19 papers for DNA sequencing. Fourth, in Dr. Wu’s first paper, only the nucleotide composition of the 5′-terminated strands of bacteriophage lambda DNA was determined with 13 nucleotides of dG, 13 of dC, 7 of dA, and 7 of dT (Wu and Kaiser, 1968). The orders of DNA sequences were not determined, and the methodology cannot be regarded as a successful sequencing approach. However, in 1970 but not 1971, Dr. Wu published a paper in *Journal of Molecular Biology* by himself, and reported a general method for determining the DNA sequence (Wu, 1970). In the abstract, he wrote: “When this terminal region is present as a single strand, as in bacteriophage lambda, *Escherichia coli* DNA polymerase can be used to repair the single-stranded region with the addition of radioactive nucleotides to the 3′-end copying the protruding 5′-terminated single strand. The partially labeled DNA can be degraded with nucleases, the radioactive oligonucleotides isolated, and their sequence determined”. By this method, he successfully determined a short sequence of the first eight of twelve nucleotides as CGCCGCCC in the right-hand protruding strand of lambda DNA (Onaga, 2014; Wu, 1970). The method was continuously improved and collectively called as the location specific-primer-extension principle, or the primer extension method (Onaga, 2014; Padmanabhan and Wu, 1972b). Taken together, we can confirm that the first approach for determining both the composition and order of DNA sequences was reported by Dr. Wu (Wu, 1970).

After Dr. Wu passed away on Feb 10th 2008, the journal of *Science in China Series C: Life Sciences* published a special issue with 12 memorial essays in 2009. One essay derived from the official obituary of Cornell University Press stated, “In 1970, Wu developed the first method for sequencing DNA and some of the fundamental tools for DNA cloning (sequencing involves determining the base sequence in a DNA molecule)” (2009). So the contribution of Dr. Wu on DNA sequencing was fairly credited, and whether we can correct his title as “father of DNA sequencing”?

OK, we assume that you will have a question: Perhaps Dr. Wu was indeed the first that started to work on DNA sequencing, but his contributions were too trivial and others did much better and more important jobs. So is it reasonable to neglect this “humble” figure? Our answer is: No. While later adaptions and applications may have a more direct impact, the fundamental principles are at the root of everything. Obviously the Sanger sequencing was developed based on Wu’s primer-extension method. If we only considered the technical innovations, we believe that Dr. Leroy Hood did the best job by developing the first automated DNA sequencer in 1986 together with Applied Biosystems. Although the Sanger sequencing was adopted in the first-generation sequencing, this approach was not used any longer in the second-generation and third-generation sequencing. However, the primer-extension principle, developed by Dr. Wu, has never been changed in all generations of sequencing techniques.

Also, we assume that you will have a second question: So whether Dr. Wu never realized his own contributions? Our answer is that he clearly knew. In an important historical research study, Prof. Lisa Onaga in Singapore analyzed and described Dr. Wu as a “Fifth Business”. The term “Fifth Business” was coined by the Canadian novelist Robertson Davies in 1970 (Onaga, 2014), to describe those who were ‘neither those of Hero nor Heroine, Confidante nor Villain, but which were nonetheless essential to bring about the Recognition or dénouement’. The lifetime and scientific careers of Dr. Wu were carefully described in details in Dr. Onaga’s article, together with Dr. Wu’s own protest on the dismissal of his seminal contributions.

So how did Dr. Wu defend himself? Prof. Lisa A. Onaga described an example. “On 11th May 2007, the journal *Science* published a colorful poster insert entitled ‘The Evolution of DNA Sequencing Technologies’. Starting with Mendel’s analysis of inheritance in plants in 1865 and ending with the 2007 announcement of the first sequence of a named human being’s genome, an unzipping double helix represented a timeline of the ‘discovery process’ leading to state-of-the-art DNA sequencing technologies” (Onaga, 2014). However, the name of Dr. Wu was not mentioned at all. So Dr. Wu felt being treated unfairly and wrote a letter to Science. Dr. Wu first acknowledged the Sanger sequencing as a great breakthrough, and continued to state the neglected fact: “However, the method was still based on my location specific-primer-extension principle in labeling the DNA before sequence analysis”. Dr. Wu insisted, “If you agree to add my contribution to the chart ‘The Evolution of Sequencing Technology,’ you may add an entry for 1970 and write something like ‘Wu introduced the first method for DNA sequence analysis by introducing the primer-extension approach.’” (Onaga, 2014). Prof. Lisa A. Onaga quoted Dr. Wu’s words verbatim, and you can directly contact her if there were any mistakes. In this regard, Dr. Wu was clearly aware of his contributions, and knew that he should be remembered as a key figure in the history of life science. He tried his best to pursue a fair recognition, although not successful.

The Human Genome Project (HGP), together with the Manhattan Project and Apollo program, were considered as the three greatest scientific projects of all time. Genome sequencing, or DNA sequencing, has had a profound impact for researches in life science and medicine, and directly spurred the emergence of multiple new fields, such as genomics, proteomics, bioinformatics/computational biology, and systems biology. So we can (perhaps arguably) regard the DNA sequencing as the most important technique in 20th century. Also, the fourth greatest project, the Precision Medicine Project, was started in 2011 with a solid foundation from HGP. Since Dr. Wu first developed the DNA sequencing methodology, and established the primer-extension principle, “father of DNA sequencing” should be a correct title for him. And his contributions can at least be comparative to Dr. Sanger, who made great contributions on determining both protein and DNA sequencing and is deserving of a title of “the emperor of sequencing”. Taken together, when we introduce the contributions of Dr. Sanger in DNA sequencing, we really have to add the information that based on the primer-extension approach developed by Dr. Wu, and Dr. Sanger further refined the methodology and played an important role in DNA sequencing. Dr. Wu’s contribution should not be and hopefully will not be neglected.

So you will have a final question: Since Dr. Wu made a great contribution, why didn’t he get the Nobel Prize? OK, we believe you must know the answer, right? If not, you can take a look at the comments from our friend, Prof. Xiaole Shirley Liu in Harvard School of Public Health:

Being a Chinese immigrant in the US in the 50’s, the social and racial challenges Ray Wu faced at that time must be tremendous. Without social backing and connections, he got where he did purely by his scientific genius and good heart. If he was a Caucasian scientist from UK or US, or even if he was in the current era, his scientific contributions would have been better recognized. Sometimes we don’t have to pay too much attention to awards or H-index, but objectively evaluate someone by their overall impact to the scientific community.

Dr. Ray Wu was born on 14th Aug, 1928 in Beijing of China. Due to the language barrier, he had to make more efforts than others when he studied in USA. Later in 1964, he listened to the talk from Robert Holley on RNA sequencing, and was greatly influenced by Max Delbrück, Alfred Hershey, and Arthur Kornberg, who made great contributions on the genetics of bacteriophages. He decided to devote to resolving the problem of DNA sequencing, succeeded six years later, raising the curtain of a splendid new era: The Genomics Era.

So for Dr. Ray Wu, the title of father of DNA sequencing is more than befitting. He well and truly deserves this recognition.

This essay is an extending discussion on an outlook published in Nature by Dr. Chuan-Chao Wang, which immediately triggered a very broad discussion among the scientific societies in China, especially to the young generation (Wang, 2015). A lot of people participated in the online conversation launched by ScienceNet. All agree with Dr. Wang’s opinion to give youth a chance. So a key question is when? We hope the time will not be 10,000 years later. We shared our viewpoints to many friends, Prof. Xiaole Shirley Liu, Prof. Liangsheng Zhang, Prof. Pengyu Huang, and Prof. Kang Ning, and are thankful for their helpful comments, which considerably improved presentation. We are also grateful for Prof. Lisa Onaga, who believes, “It is really interesting to know how the Chinese memory of Dr. Wu is quite different. Your comment reinforces my understanding that there is a different picture of this individual and I hope to continue to do more research to better understand the later part of his career” (Personal communication, 16th August, 2015). We also thank Prof. Le Kang for his encouragement, and Dr. Xiaoxue Zhang of *Protein* & *Cell* Editorial Office, for her kindness and patience. Yu Xue wrote the blog in Chinese by himself, but his student Yongbo Wang and his friend Prof. Hui Shen made great efforts on drafting and refining the English version.

Finally, it’s the first time for Yu Xue to write an essay but not a formal scientific paper in English, or Chinglish. He used to write in Chinese, and is not sure whether Chinese and English-speaking people have a different sense of humor. When he attended conferences, a lot of foreign scientists frequently laughed at certain jokes the speakers were telling, when he was totally lost and confused. He pretended to laugh as well, of course, but in that light his efforts of being witty or humorous at certain part of the article might have been a total failure. We apologize for that, but hopefully it will not affect the message that we are trying to convey.
